# Clinical Outcomes of First-Time and Redo Mitral Valve Replacement Using MITRIS RESILIA Bioprosthesis

**DOI:** 10.1016/j.atssr.2025.12.015

**Published:** 2026-01-10

**Authors:** Satoshi Kainuma, Tomoki Shimokawa, Tomohiro Iwakura, Haruki Mikoshiba, Atsushi Yano, Atsushi Kurushima, Tatsuo Motoki, Yoshiaki Fukumura, Kazuhisa Sakamoto, Yuki Wada, Nobuhisa Ohno, Tomoki Ushijima, Akira Shiose, Katsuhiro Omae, Manabu Minami, Haruko Yamamoto, Rieko Kutsuzawa, Shinya Tajima, Tomoyuki Fujita, Satsuki Fukushima

**Affiliations:** 1 Department of Cardiovascular Surgery, National Cerebral and Cardiovascular Center, Osaka, Japan; 2 Sakakibara Heart Institute, Tokyo, Japan; 3 Teikyo University, Tokyo, Japan; 4 Japanese Red Cross Tokushima Hospital, Tokushima, Japan; 5 Kokura Memorial Hospital, Fukuoka, Japan; 6 Hamamatsu Rosai Hospital, Shizuoka, Japan; 7 Kyushu University Hospital, Fukuoka, Japan; 8 Department of Data Science, National Cerebral and Cardiovascular Research Center, Osaka, Japan; 9 Graduate School of Medical and Dental Sciences, Institute of Science Tokyo, Tokyo, Japan

## Abstract

**Background:**

The MITRIS RESILIA bioprosthetic valve incorporates anticalcification technology and is designed to facilitate implantation in fragile mitral annuli. However, clinical data on its safety and hemodynamic performance remain limited.

**Methods:**

We conducted a retrospective multicenter study of 140 patients (mean age, 75 ± 8 years; 44% male) who underwent first-time (n = 100) or redo (n = 40) mitral valve replacement (MVR) with a MITRIS RESILIA valve between 2021 and 2022. The primary indication for first-time MVR was mitral regurgitation, followed by mitral stenosis, whereas that for redo MVR was prosthetic valve dysfunction, followed by failed repair. The mean follow-up duration was 36 ± 14 months (415 patient-years).

**Results:**

The most frequently implanted valve size was 27 mm (31%), followed by 29 mm (26%) and 25 mm (24%). Thirty-day mortality was 2.0% for first-time MVR patients and 0% for redo patients. At 3 years, freedom from all-cause mortality, reoperation, stroke, and structural valve deterioration was 89%, 99%, 96%, and 100%, respectively, with no intergroup differences. The mixed effects model found stable and favorable prosthetic hemodynamics over time—reflected by mean pressure gradients, effective orifice area, and the absence of major paravalvular leakage. Both groups showed sustained reductions in left atrial size and tricuspid regurgitation pressure gradient, accompanied by parallel improvements in New York Heart Association functional class (all time effect *P* < .001).

**Conclusions:**

First-time and redo MVR with the MITRIS RESILIA valve was associated with low perioperative mortality, favorable safety, and durable hemodynamic performance. These findings support its use across a broad spectrum of mitral valve diseases.

**In Short**

▪ First-time and redo mitral valve replacement with the MITRIS RESILIA valve was associated with low perioperative mortality, favorable safety, and durable hemodynamic performance.
▪ These findings support the use of the MITRIS RESILIA valve across a broad spectrum of mitral valve diseases.

Mitral valve replacement (MVR) remains an essential option for patients with symptomatic mitral valve disease who are unsuitable for durable repair, including those with complex degenerative or functional disease, recurrent regurgitation, significant stenosis, or prior valve surgery.^1^ The use of bioprosthetic valves has increased markedly during the past 2 decades, emphasizing the importance of prosthesis selection.^2^ Bioprosthetic valves offer important advantages, including the avoidance of lifelong anticoagulation and a lower risk of bleeding complications.^1^ Although bioprostheses avoid lifelong anticoagulation and reduce bleeding risk, MVR continues to carry higher perioperative mortality than mitral repair, particularly in elderly and redo patients with compromised annular tissue. Their long-term durability is further limited by structural valve deterioration (SVD), often necessitating reoperation.

The MITRIS RESILIA valve (Edwards Lifesciences), introduced in Japan in 2021, combines the design of the Carpentier-Edwards PERIMOUNT Magna Mitral Ease valve with RESILIA tissue technology, an advanced anticalcification treatment aimed at improving durability.^3^ Although favorable outcomes with RESILIA tissue have been reported, contemporary clinical evidence specific to surgical MVR remains limited.^4^ This multicenter study evaluated the early and midterm safety and hemodynamic performance of the MITRIS RESILIA valve in patients undergoing primary or redo MVR.

## Patients and Methods

This nonrandomized, retrospective multicenter study included 140 adults who underwent first-time or redo MVR with the MITRIS RESILIA bioprosthesis at 5 institutions in Japan between April 2021 and April 2022. Patients were assigned to 2 groups: a first-time MVR group, comprising individuals undergoing initial MVR regardless of prior cardiac surgery; and a redo MVR group, comprising those undergoing repeated MVR after prior mitral repair or replacement. Detailed methodologic information is provided in the Supplemental Material.

## Results

### Baseline Clinical Characteristics

Details of baseline demographics, surgical data, and early outcomes are summarized in Supplemental Tables 1 and 2. Mean patient age was 75 ± 7.6 years, and 44% were male; mean body surface area was 1.51 ± 0.17 m^2^. A total of 46 (33%) previously underwent prior cardiac surgery, most commonly mitral valve repair (n = 10) or replacement (n = 30); the remainder underwent aortic valve replacement or atrial septal defect closure. Of these, 40 composed the redo-MVR group and the remaining 100 the first-time MVR group. The predominant pathologic process in the first-time group was mitral regurgitation, followed by mitral stenosis; in the redo MVR group, procedures were most often indicated for prosthetic valve failure, repair failure, progression of mitral stenosis after commissurotomy, or perivalvular leak.

### Surgical Data

The most frequently chosen valve size was 27 mm (31%), followed by 29 mm (26%) and 25 mm (24%). An isolated MVR procedure was performed in nearly one-fourth of the patients (n = 32 [23%]); the remaining 77% underwent MVR with a concomitant procedure. Patients who underwent primary MVR were more likely to undergo a concomitant procedure. However, there were no intergroup differences for operation, cardiopulmonary bypass, or aortic cross-clamp time or in length of hospital stay.

### Safety Outcomes

The 30-day and hospital mortality rates were 1.4% (n = 2) and 3.6% (n = 5), respectively. Each of the 5 with mortality underwent primary MVR with a concomitant procedure; at the baseline examination, 3 were dependent on inotropic agents and 4 presented with low left ventricular (LV) ejection fractions. The leading cause of hospital mortality was low output syndrome (n = 3), followed by LV rupture (n = 1) and pneumonia (n = 1). Within 30 days after surgery, 3 experienced an ischemic stroke, whereas none underwent a reoperation or showed endocarditis or valve dysfunction.

**Figure 1:**
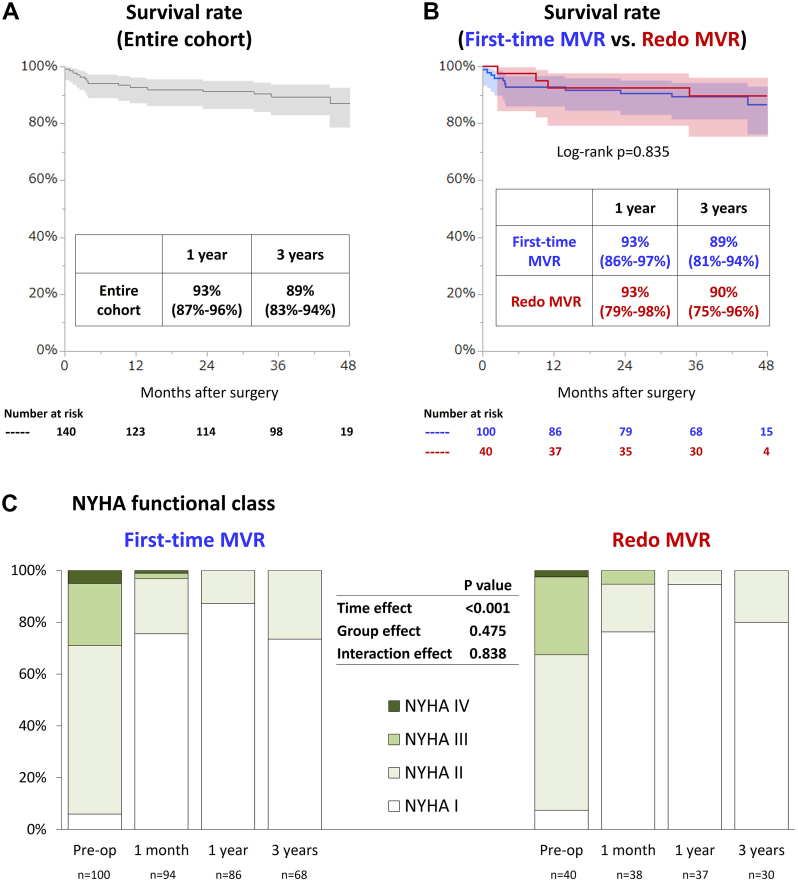
(A) Overall survival rate after mitral valve replacement (MVR) with MITRIS RESILIA valve. (B) Overall survival rate after MVR by history of mitral surgery. (C) Serial changes in New York Heart Association (NYHA) functional class after first-time and redo MVR. (Pre-op, preoperative.)

The mean follow-up period was 36 ± 14 months (415 patient-years). Within 3 years, there were 14 deaths (10 in first-time MVR), 1 reoperation for valve thrombosis (first-time MVR), and 6 cases of stroke (5 in first-time MVR); there were no cases of endocarditis, major paravalvular leak, SVD, or non-SVD. The 3-year survival rate was 89% (83%-94%), with no significant intergroup differences (Figures 1A, 1B). Likewise, 3-year freedom from reoperation, stroke, and SVD was 99% (95%-100%), 96% (90%-98%), and 100% (100%-100%), respectively, without significant intergroup differences (Table).

**Table.** Early and Three-Year Outcomes of First-Time or Redo MVR

Outcome	Early (<30 Days), No. (%)		Cumulative at 3 Years, No. (%)		Probability Event-Free at 3 Years,% (95% CI)		*P* Value
	First-Time MVR(n = 100)	Redo MVR(n = 40)	First-Time MVR(n = 100)	Redo MVR(n = 40)	First-Time MVR(n = 100)	Redo MVR(n = 40)	
All-cause mortality	2 (2.0)	0 (0)	10 (10)	4 (10)	89 (81-94)	90 (75-96)	.835
Reoperation	0 (0)	0 (0)	1 (1.0)	0 (0)	99 (92-100)	100 (100-100)	.509
Stroke	3 (3.0)	0 (0)	5 (5.0)	1 (2.5)	95 (88-98)	97 (83-100)	.479
Endocarditis	0 (0)	0 (0)	0 (0)	0 (0)	100 (100-100)	100 (100-100)	1
Valve thrombosis	0 (0)	0 (0)	1 (1.0)	0 (0)	99 (92-100)	100 (100-100)	.509
Major PVL^a^	0 (0)	0 (0)	0 (0)	0 (0)	100 (100-100)	100 (100-100)	1
SVD	0 (0)	0 (0)	0 (0)	0 (0)	100 (100-100)	100 (100-100)	1
Non-SVD	0 (0)	0 (0)	0 (0)	0 (0)	100 (100-100)	100 (100-100)	1

MVR, mitral valve replacement; PVL, paravalvular leak; SVD, structural valve deterioration.

a Major PVL: paravalvular leak of any grade requiring surgical intervention or considered a serious adverse event.

### Functional Status

New York Heart Association functional class at baseline was I, II, III, and IV in 9 (6.4%), 89 (64%), 36 (26%), and 6 (4.3%) patients, respectively. That classification was significantly improved after 1 month and remained sustained for up to 3 years after surgery, with no intergroup differences (time effect *P* < .001; interaction effect *P* = .838; Figure 1C).

### Hemodynamic Outcomes

Postoperative serial echocardiographic results are summarized in Supplemental Table 3. From baseline to 1 week after surgery, LV end-diastolic dimension was decreased, LV systolic dimension did not change, and LV ejection fraction decreased in both groups (Supplemental Figures A-C). LA dimension and tricuspid regurgitation pressure gradient substantially decreased up to 1 year after surgery and thereafter remained or slightly increased (Supplemental Figures D, E). From 1 week to 3 years, LV end-systolic dimension was decreased, whereas LV ejection fraction improved, and these improvements were generally sustained up to 3 years in both groups.

**Figure 2:**
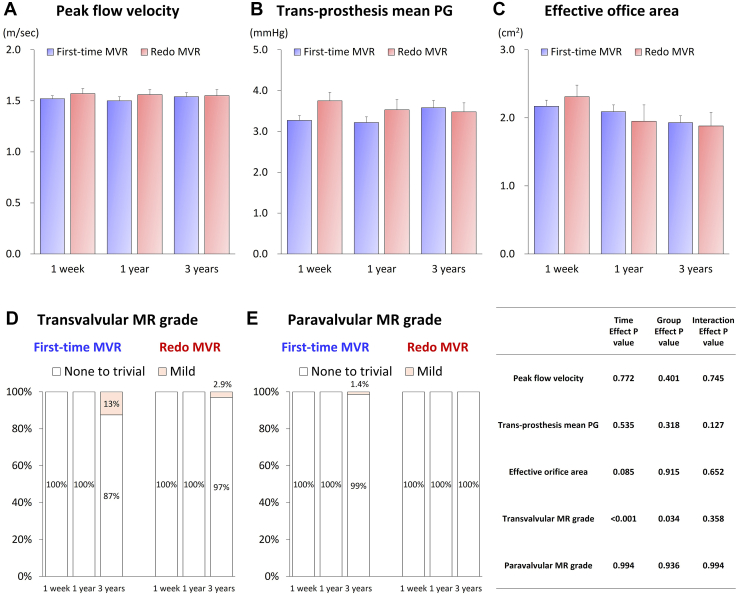
Serial postoperative prosthetic hemodynamics after first-time or redo mitral valve replacement (MVR). (A) Peak flow velocity. (B) Trans-prosthesis mean pressure gradient (PG). (C) Effective orifice area. (D) Transvalvular mitral regurgitation (MR) grade. (E) Paravalvular MR grade.

**Figure 3:**
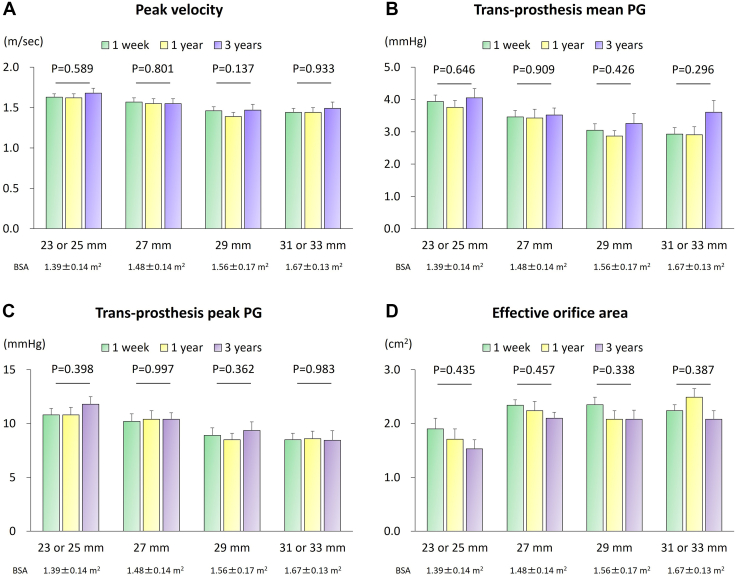
Serial postoperative (A) peak flow velocity, (B) trans-prosthesis mean pressure gradient (PG), (C) trans-prosthesis peak PG, and (D) effective orifice area after mitral valve replacement according to prosthetic valve size. (BSA, body surface area.)

In addition, each group exhibited stable peak mitral velocity, mean and peak pressure gradient, and effective orifice area findings, without major transvalvular or paravalvular leaks, indicating favorable prosthetic hemodynamics (Figure 2). Notably, at 1 week after MVR with a 23- or 25-mm MITRIS valve, peak velocity, mean and peak pressure gradients, and effective orifice area were 1.6 ± 0.3 m/s, 3.9 ± 1.3 mm Hg, 11 ± 3.5 mm Hg, and 1.9 ± 0.9 cm^2^, respectively, and remained unchanged for up to 3 years (time effect *P* > .05 for all; Figure 3).

## Comment

This multicenter study found that MVR with a MITRIS RESILIA valve for both first-time and redo cases was safe and associated with stable hemodynamic performance up to 3 years. Furthermore, hospital mortality was relatively low, despite advanced patient age and frequent concomitant procedures. Importantly, no SVD, endocarditis, or clinically significant paravalvular leak was observed, underscoring the favorable safety profile of this prosthesis.

The principal concern remaining for MVR is relatively high hospital mortality. Mortality also remains substantial for higher risk subgroups, reaching 13.9% for MVR combined with coronary artery bypass grafting, 5.1% for MVR with tricuspid valve surgery, and 6.5% for redo MVR.^5^ In contrast, hospital mortality rates for this cohort were 0% for isolated MVR and 4.6% for procedures performed with concomitant surgery, both notably lower than reported in national registry data. Furthermore, redo MVR in this series was not associated with in-hospital mortality, irrespective of concomitant intervention. Moreover, early safety outcomes, including reoperation, stroke, endocarditis, and valve dysfunction, were within acceptable limits and comparable to those reported in the COMMENCE trial, despite this cohort’s having an older average age (75 vs 70 years) and a greater prevalence of concomitant procedures (77% vs 57%).^4^

Several features of the MITRIS RESILIA valve, such as a low-profile design that minimizes the risk of LV outflow tract obstruction and a pliable saddle-shaped sewing cuff that facilitates secure implantation in a fragile or previously operated on annulus, may have contributed to the low hospital mortality rate and few adverse procedure-related events. The low-profile design is beneficial for patients with a significantly hypertrophic left ventricle and a narrow LV outflow tract as well as for those who undergo double valve replacement, which accounted for nearly 30% of the cases examined in this study. As for the pliable saddle-shaped sewing cuff, no clinically significant paravalvular leakage was detected, regardless of a history of previous mitral surgery, suggesting that the MITRIS valve is beneficial for patients with weakened annular tissues and for those in whom residual foreign material might interfere with the healing of the new prosthesis-annular interface.

In line with the US COMMENCE trial findings, the most frequently implanted prosthetic valve size in these patients was 27 mm, followed by 29 mm.^4^ However, use of a smaller prosthesis (23-25 mm) was markedly more common in this cohort than in the COMMENCE trial (27% vs 7.3%), likely reflecting differences in average body size between Japanese and Western populations. The present findings also indicate that prosthetic valve performance was favorable, with stable mean pressure gradient and peak flow velocity and minimal paravalvular or transvalvular regurgitation, consistent with prior reports. Notably, the relatively low mean gradients observed after implantation of 23- or 25-mm valves support their applicability in patients with smaller body surface area. In addition, comparable improvements in ventricular and atrial remodeling, transvalvular gradient, and functional class were observed in both the first-time and redo groups, suggesting consistent performance across operative complexity and annular quality.

### Limitations

Although the follow-up period was short, the absence of SVD and valve-related reoperation cases is encouraging. Longer surveillance, particularly beyond 5 years, is necessary to confirm durability. Nevertheless, these findings support the MITRIS RESILIA valve as a reliable option for both first-time and redo MVR for a broad range of mitral valve diseases.

### Conclusion

Our data demonstrated a favorable safety profile and clinically stable hemodynamic performance of the MITRIS RESILIA valve in patients requiring replacement of the native or prosthetic mitral valve, with or without concomitant procedures. The MVR with the MITRIS RESILIA mitral valve is therefore considered the treatment of choice for a wide range of mitral valve diseases.

## Figures and Tables

**Supplementary Figure 1:**
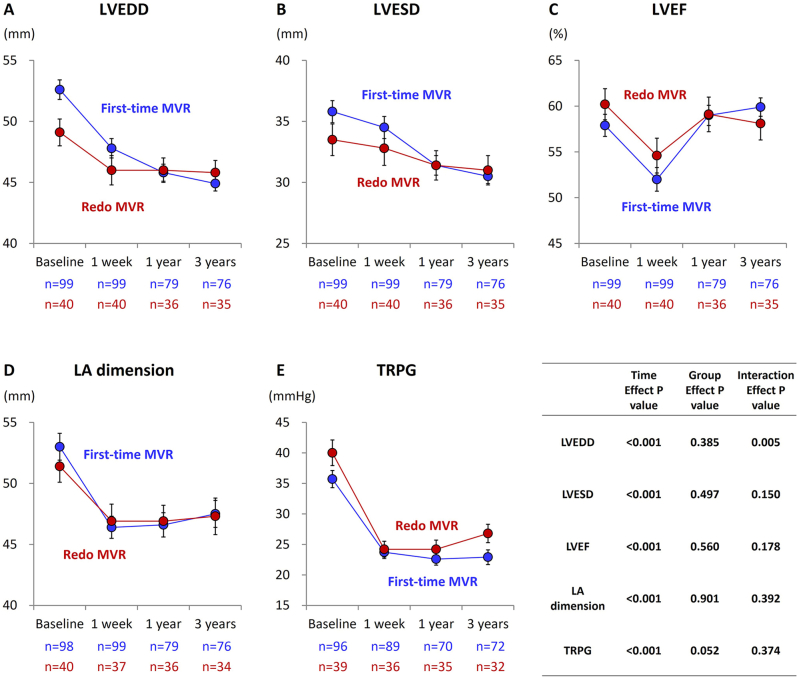
Serial changes in (A) LVEDD, (B) LVESD, (C) LVEF, (D) LA dimension, and (E) TRPG following first-time and redo MVR. Data are presented as mean ± standard error. (LA, left atrial; LVEDD, left ventricular end-diastolic dimension; LVEF, left ventricular ejection fraction; LVESD, left ventricular end-systolic dimension; TRPG, tricuspid regurgitation pressure gradient.)
