# Supplementary material for: Clinical Outcomes of First-Time and Redo Mitral Valve Replacement Using MITRIS RESILIA Bioprosthesis
Source: Ann Thorac Surg Short Rep. 2026 Jan 10;4(2):618–24. doi: 10.1016/j.atssr.2025.12.015 (PMC13245502; doi:10.1016/j.atssr.2025.12.015)
Supplement: Supplementary Material [file mmc1.doc]

**Patients and Methods**

**Materials and methods**

For this non-randomized multicenter observational study, clinical outcomes of 154 adult patients who underwent first-time or redo MVR with a MITRIS RESILIA biological valve at five institutions in Japan between April 2021 and April 2022 were retrospectively reviewed. Those with a concomitant tricuspid valve repair, aortic valve replacement, coronary artery bypass grafting, or ablation surgery procedure were included. A total of 14 patients with either shock while on mechanical support, a required extracardiac procedure, or active infective endocarditis within the past three months, or with abnormal calcium metabolism or hyperparathyroidism were excluded. Finally, 140 patients who underwent MVR were enrolled (mean age, 75±8 years; body surface area, 1.51±0.17 m²). Data regarding clinical characteristics, surgical data, and outcomes were obtained from the surgical database of each participating institution. This observational study was approved by the Ethics Committee of the National Cerebral and Cardiovascular Center based on a centralized review (reference no. R22042-5). Patients were allowed to decline participation if they did not wish to be included.

***Surgical device and intervention procedures***

Surgical MVR was performed using a MITRIS RESILIA mitral valve (Edwards Lifesciences, Irvine, CA), commercially introduced in April 2021. The surgical approach and implantation technique were performed at the discretion of the primary surgeon in each case. Patients routinely received oral warfarin for three to six months after surgery. Thereafter, maintenance of anticoagulation therapy was determined on an individual basis according to risk of thromboembolism and cardiac rhythm status. The surgeon in charge of the case made the final decision to start and stop antiplatelet and/or anticoagulant therapy.

***Postoperative management***

All patients received anticoagulation therapy with warfarin during the first three to six months postoperatively, unless contraindicated, and the medication was discontinued if the patients remained in sinus rhythm. If they had any evidence of intermittent or persistent atrial fibrillation, warfarin was continued or changed to a novel oral anticoagulant. Thereafter, maintenance of anticoagulation therapy was determined on an individual basis, taking into account the risk of thromboembolism and the patient’s cardiac rhythm status. However, the final decision to stop or start anticoagulant therapy was made at the discretion of the primary surgeon or physician in each case.

***Safety endpoints***

Safety endpoints were evaluated in the early postoperative period (≤30 days) and again at three years after surgery, and included all-cause mortality, reoperation, stroke, endocarditis, valve thrombosis, major paravalvular leak, structural valve dysfunction (SVD), and non-SVD. SVD was deﬁned as dysfunction or deterioration involving the treated valve (exclusive of infection or thrombosis), as determined from reoperation, autopsy, or clinical investigation findings. Conversely, non-SVD was deﬁned as any abnormality that did not directly involve valve components yet resulted in dysfunction of the treated valve or hemolysis.

***Assessments of hemodynamics and functional factors***

Serial two-dimensional and Doppler echocardiography examinations were performed preoperatively (baseline) to evaluate longitudinal changes in left ventricular (LV) function parameters, left atrial dimension, and tricuspid regurgitation pressure gradient, then again at discharge, one year, and three years after surgery. For evaluation of hemodynamic performance of the prosthetic valve, peak velocity, mean mitral pressure gradient, and severity of valve regurgitation were used. Paravalvular or transvalvular regurgitation was graded as none, mild, moderate, moderate to severe, or severe. Functional status was assessed according to the New York Heart Association (NYHA) criteria for symptoms of heart failure preoperatively (baseline), one month, one year, and three years after surgery.

***Group classification***

Patients were classified into two groups based on whether they had undergone mitral surgery before MVR with a MITRIS RESILIA biological valve; those who underwent an initial MVR regardless of history of open heart surgery (first-time MVR group), and those with subsequent MVR performed after previously undergoing a mitral valve repair or replacement procedure (redo MVR group).

***Statistical analysis***

Continuous variables are presented as the mean ± standard deviation, and categorical variables as frequency and proportion. For continuous variables, comparisons were made using Student’s t-test, while chi-square analysis or Fisher’s exact test was used for comparisons of categorical variables. Echocardiographic and functional variables over time were analyzed using a linear mixed-effects model for repeated measures. The model included fixed effects for group, time (treated as a categorical variable), and their interaction (group × time). Random effects accounted for the within-subject correlation by including a random intercept for each patient. The variance-covariance structure of the repeated measurements was assumed to be unstructured, allowing for flexible modeling of within-subject variability.

Early and late safety events, deﬁned as occurring ≤30 days after the index procedure and three years after surgery, respectively, are presented as number of events divided by number of enrolled patients. In addition, Kaplan-Meier estimates of event-free survival were calculated throughout the follow-up period, with a log-rank test used to compare the patient groups. Statistical analyses were performed using JMP Pro, version 15.1.0 (SAS Institute Inc., Cary, NC, USA).

| Supplemental Table 1. Patient demographics and echocardiographic data at baseline | | | | |
| --- | --- | --- | --- | --- |
|  | All (n=140) | First-time MVR (n=100) | Redo MVR (n=40) | P value |
| Demographics |  |  |  |  |
| Age | 75±7.6 | 75±6.6 | 75±10 | 0.816 |
| Male | 61 (44%) | 45 (45%) | 16 (40%) | 0.589 |
| Body surface area, m^2^ | 1.51±0.17 | 1.52±0.17 | 1.47±0.17 | 0.140 |
| NYHA III/IV | 42 (30%) | 29 (29%) | 13 (33%) | 0.684 |
| Diabetes | 21 (15%) | 16 (16%) | 5 (13%) | 0.595 |
| Hyperlipidemia | 37 (26%) | 23 (23%) | 14 (35%) | 0.153 |
| Hemodialysis | 5 (3.6%) | 5 (5.0%) | 0 (0%) | 0.321 |
| COPD | 29 (21%) | 22 (22%) | 7 (18%) | 0.548 |
| Post-open heart surgery | 46 (33%) | 6 (6.0%) | 40 (100%) | <0.001 |
| Cardiac rhythm |  |  |  |  |
| Sinus | 63 (45%) | 41 (41%) | 22 (55%) | 0.026 |
| Atrial arrhythmia | 68 (49%) | 55 (55%) | 13 (33%) |  |
| Pacing rhythm | 9 (6.4%) | 4 (4.0%) | 5 (13%) |  |
| Surgical indication |  |  |  |  |
| Mitral regurgitation | 69 (49%) | 69 (69%) | - | NA |
| Mitral stenosis | 27 (19%) | 27 (27%) | - |  |
| Others | 4 (2.9%) | 4 (4.0%) | - |  |
| Prosthetic valve failure | 25 (18%) | - | 25 (63%) |  |
| Perivalvular leak | 2 (1.4%) | - | 2 (5.0%) |  |
| Post OMC | 3 (2.0%) | - | 3 (7.5%) |  |
| Post-mitral repair | 10 (7.1%) | - | 10 (25%) |  |
| Echocardiographic data |  |  |  |  |
| LVEDD, mm | 52±8.0 | 53±8.4 | 49±7.0 | 0.022 |
| LVESD, mm | 35±8.7 | 36±8.9 | 34±8.0 | 0.174 |
| LA dimension, mm | 52±11 | 53±12 | 51±8.2 | 0.449 |
| LV ejection fraction, % | 59±12 | 58±12 | 60±11 | 0.295 |
| >Moderate grade AS | 16 (12%) | 14 (14%) | 2 (5.1%) | 0.102 |
| >Moderate grade AR | 11 (8.0%) | 8 (8.2%) | 3 (7.7%) | 0.927 |
| >Moderate grade TR | 30 (21%) | 21 (21%) | 9 (23%) | 0.890 |
| Abbreviations: MVR = mitral valve replacement, NYHA = New York Heart Association functional classification, COPD = chronic obstructive pulmonary disease, OMC = open mitral commissurotomy, PT-INR = prothrombin time and international normalized ratio, LVEDD = left ventricular end-diastolic dimension, LVESD = left ventricular end-systolic dimension, LA = left atrial, LV = left ventricular, AS = aortic stenosis, AR = aortic regurgitation, TR = tricuspid regurgitation | | | | |

| Supplemental Table 2. Surgical data and early outcomes | | | | |
| --- | --- | --- | --- | --- |
|  | All (n=140) | First-time MVR (n=100) | Redo MVR (n=40) | P value |
| Prosthetic valve size, n (%) |  |  |  |  |
| 23 mm | 4 (2.9%) | 2 (2.0%) | 2 (5.0%) | 0.196 |
| 25 mm | 34 (24%) | 24 (24%) | 10 (20%) |  |
| 27 mm | 44 (31%) | 28 (28%) | 16 (40%) |  |
| 29 mm | 36 (26%) | 26 (26%) | 10 (25%) |  |
| 31 mm | 21 (15%) | 19 (19%) | 2 (5.0%) |  |
| 33 mm | 1 (0.7%) | 1 (1.0%) | 0 (0%) |  |
| Procedures |  |  |  |  |
| Isolated MVR | 32 (23%) | 11 (11%) | 21 (53%) | <0.001 |
| MVR with concomitant surgery | 108 (77%) | 89 (89%) | 19 (47%) |  |
| Concomitant surgery, n (%) |  |  |  |  |
| TAP | 78 (56%) | 65 (65%) | 13 (33%) | <0.001 |
| AVR | 41 (29%) | 34 (34%) | 7 (18%) | 0.045 |
| CABG | 19 (14%) | 18 (18%) | 1 (2.5%) | 0.006 |
| Ablation surgery | 36 (26%) | 33 (33%) | 3 (7.5%) | 0.001 |
| LAA clip | 67 (48%) | 61 (61%) | 6 (15%) | <0.001 |
| Morrow operation | 1 (0.7%) | 1 (1.0%) | 0 (0%) | 1.000 |
| Ascending aorta replacement | 1 (0.7%) | 1 (1.0%) | 0 (0%) | 1.000 |
| Operation time, mins |  |  |  |  |
| Isolated MVR (n=32) | 254±72 | 236±84 | 263±64 | 0.321 |
| Concomitant procedure (n=108) | 325±110 | 318±108 | 360±117 | 0.135 |
| CPB time, mins |  |  |  |  |
| Isolated MVR (n=32) | 128±53 | 128±67 | 128±43 | 0.977 |
| Concomitant procedure (n=108) | 182±67 | 181±61 | 188±90 | 0.679 |
| ACC time, mins |  |  |  |  |
| Isolated MVR (n=32) | 84±37 | 88±53 | 83±25 | 0.719 |
| Concomitant procedure (n=108) | 130±46 | 129±43 | 133±57 | 0.747 |
| Early outcomes |  |  |  |  |
| 30-day mortality, n (%) | 2 (1.4%) | 2 (2.0%) | 0 (0%) | 1.000 |
| Isolated MVR (n=32) | 0 (0%) | 0 (0%) | 0 (0%) | 1.000 |
| Concomitant procedure (n=108) | 2 (1.9%) | 2 (2.3%) | 0 (0%) | 1.000 |
| Hospital mortality, n (%) | 5 (3.6%) | 5 (5.0%) | 0 (0%) | 0.321 |
| Isolated MVR (n=32) | 0 (0%) | 0 (0%) | 0 (0%) | 1.000 |
| Concomitant procedure (n=108) | 5 (4.6%) | 5 (5.6%) | 0 (0%) | 0.584 |
| Hospital stay, days | 24±19 | 24±17 | 24±22 | 0.881 |
| Abbreviations: MVR = mitral valve replacement, TAP = tricuspid annuloplasty, AVR = aortic valve replacement, CABG = coronary artery bypass grafting, LAA = left atrial appendage, CPB = cardiopulmonary bypass, ACC = aortic cross-clamp | | | | |

| Supplemental Table 3. Postoperative echocardiographic data | | | | |
| --- | --- | --- | --- | --- |
|  | Entire (n=140) | First-time MVR (n=100) | Redo MVR (n=40) | p value |
| Echocardiography at 1 week | n=139 | n=99 | n=40 |  |
| LVEDD, mm | 47±8.0 | 48±8.1 | 46±7.7 | 0.212 |
| LVESD, mm | 34±8.7 | 35±8.7 | 33±8.8 | 0.281 |
| LVEF, % | 53±13 | 52±13 | 55±12 | 0.301 |
| LA dimension, mm | 47±8.8 | 46±8.8 | 47±8.7 | 0.764 |
| TRPG, mmHg | 24±8.8 | 24±9.2 | 24±7.8 | 0.767 |
| Peak velocity (m/sec) | 1.5±0.3 | 1.5±0.3 | 1.6±0.3 | 0.419 |
| Mean PG, mmHg | 3.4±1.3 | 3.3±1.2 | 3.7±1.3 | 0.060 |
| Peak PG, mmHg | 9.8±3.9 | 9.6±3.6 | 10±4.7 | 0.428 |
| EOA, cm^2^ | 2.2±0.7 | 2.2±0.7 | 2.3±0.7 | 0.465 |
| Transvalvular regurgitation >mild, n (%) | 0 (0%) | 0 (0%) | 0 (0%) | 1.000 |
| Perivalvular regurgitation >mild, n (%) | 0 (0%) | 0 (0%) | 0 (0%) | 1.000 |
| Echocardiography at 1 year | n=119 | n=83 | n=36 |  |
| LVEDD, mm | 45±7.2 | 45±7.6 | 46±6.3 | 0.609 |
| LVESD, mm | 31±7.4 | 31±7.3 | 31±7.5 | 0.851 |
| LVEF, % | 58±11 | 58±11 | 59±11 | 0.658 |
| LA dimension, mm | 47±8.5 | 46±8.9 | 47±7.5 | 0.764 |
| TRPG, mmHg | 23±8.4 | 23±8.2 | 24±8.9 | 0.402 |
| Peak velocity (m/sec) | 1.5±0.3 | 1.5±0.3 | 1.6±0.3 | 0.372 |
| Mean PG, mmHg | 3.3±1.3 | 3.2±1.2 | 3.5±1.4 | 0.237 |
| Peak PG, mmHg | 9.7±4.0 | 9.6±4.2 | 10±3.5 | 0.550 |
| EOA, cm^2^ | 2.1±0.7 | 2.1±0.7 | 2.0±0.7 | 0.626 |
| Transvalvular regurgitation >mild, n (%) | 0 (0%) | 0 (0%) | 0 (0%) | 1.000 |
| Perivalvular regurgitation >mild, n (%) | 0 (0%) | 0 (0%) | 0 (0%) | 1.000 |
| Echocardiography at 3 years | n=106 | n=72 | n=34 |  |
| LVEDD, mm | 45±5.3 | 45±5.6 | 45±4.8 | 0.924 |
| LVESD, mm | 31±5.5 | 31±5.6 | 30±5.4 | 0.694 |
| LVEF, % | 60±9.2 | 60±8.8 | 59±10 | 0.616 |
| LA dimension, mm | 48±9.0 | 48±9.2 | 47±8.7 | 0.855 |
| TRPG, mmHg | 25±9.4 | 23±9.7 | 27±8.7 | 0.080 |
| Peak velocity (m/sec) | 1.6±0.4 | 1.6±0.3 | 1.6±0.4 | 0.897 |
| Mean PG, mmHg | 3.6±1.5 | 3.7±1.6 | 3.4±1.3 | 0.361 |
| Peak PG, mmHg | 10±4.1 | 10±4.1 | 11±4.0 | 0.600 |
| EOA, cm^2^ | 1.9±0.6 | 1.9±0.6 | 1.9±0.6 | 0.808 |
| Transvalvular regurgitation >mild, n (%) | 0 (0%) | 0 (0%) | 0 (0%) | 1.000 |
| Perivalvular regurgitation >mild, n (%) | 0 (0%) | 0 (0%) | 0 (0%) | 1.000 |
| Abbreviations: LVEDD = left ventricular end-diastolic dimension, LVESD = left ventricular end-systolic dimension, LVEF = left ventricular ejection fraction, LA = left atrial, TRPG = tricuspid regurgitation peak gradient, PG = pressure gradient, EOA = effective orifice area | | | | |

**Supplemental Figure 1.** Serial changes in LVEDD, LVESD, LV ejection fraction, LA dimension, and TRPG following first-time and redo MVR. Data are presented as mean ± standard error. The table shows P values from linear mixed-effects model analysis for three effects: previous mitral surgery (Group effect), time (Time effect), and previous mitral surgery × time (Interaction effect) for each parameter.

Abbreviations: LVEDD = left ventricular end-diastolic dimension, LVESD = left ventricular end-systolic dimension, LV = left ventricular, LA = left atrial, TRPG = tricuspid regurgitation pressure gradient
